# Hollow Co_3_O_4_@MnO_2_ Cubic Derived From ZIF-67@Mn-ZIF as Electrode Materials for Supercapacitors

**DOI:** 10.3389/fchem.2019.00831

**Published:** 2019-12-13

**Authors:** Jiani Xu, Chaoting Xu, Yanhong Zhao, Jianghong Wu, Junqing Hu

**Affiliations:** ^1^College of Health Science and Environmental Engineering, Shenzhen Technology University, Shenzhen, China; ^2^State Key Laboratory for Modification of Chemical Fibers and Polymer Materials, College of Materials Science and Engineering, Donghua University, Shanghai, China; ^3^College of Materials Science and Engineering, Changsha University of Science & Technology, Changsha, China

**Keywords:** metal-organic frameworks, ZIF-67, Mn-ZIF, Co_3_O_4_@MnO_2_, supercapacitors

## Abstract

Hollow Co_3_O_4_@MnO_2_ cubic nanomaterials are synthesized by ZIF-67@Mn-ZIF sacrificial precursor through a facile thermal treatment. As a kind of supercapacitor electrode material, it demonstrates high performances, such as specific capacitance of 413 F g^−1^ at the current density of 0.5 A g^−1^; as the current densities raised from 0.5 to 10 A g^−1^ (20 times increasing), there is still ~41% retention of its initial capacitance. These satisfactory electrochemical properties should be put down to the hollow and porous structure and the relative higher BET surface area, which supplies more reactive sites for charge and discharge processes.

## Introduction

As a new and efficient energy storage device, supercapacitors qualified the benefits of high-power density, high security, long service life, and fast short time storage and release (El-Kady et al., 2016; Shao et al., 2018; Xu et al., 2018). As a result, supercapacitors attracted wide attention in the application on portable consumer electrical products and electric automobiles, and so on (Qu et al., 2016; Li et al., 2018). The performance of the used electrode materials is the main factor affecting the performance of supercapacitors. Currently, the most studied materials are carbon-based materials (Zhang and Zhao, 2009; Zhang et al., 2017), transition metal oxides (TMOs) (Liu et al., 2011; Li et al., 2014; Yu and Lou, 2018; Xu et al., 2019), and conductive polymer materials (Snook et al., 2011; Du et al., 2017). In recent years, metal-organic frameworks (MOFs) are developed as a new type of porous materials ascribed to their great specific surface area, porosity and regulatory pores, functional and special optical and electrical properties (Yue et al., 2015; Salunkhe et al., 2017). So they have great potential in the high-performance supercapacitor after thermal treatment as TMOs' sacrificial precursor.

Up to the present, numerous TMO nanomaterials have been synthesized as supercapacitor electrodes from many kinds of MOF precursors. For instance, high surface area Co_3_O_4_ nanoparticles have been obtained from the pyrolysis of ZIF-67 with an appreciable 190 F g^−1^ specific capacitance value at 5 A g^−1^ (Saraf et al., 2019), NiO architecture with porous structure was constructed by thermal treatment Ni-MOF under the air flow and demonstrated 324 F g^−1^ at 1 A g^−1^ (Han et al., 2017), and porous hollow α-Fe_2_O_3_ microboxes synthesized by using MOF as precursor and self-template can reach 380 F g^−1^ at 0.1 A g^−1^ as supercapacitor electrode (Yu et al., 2019). Except for these single metal oxides, some mixed metal oxides, and metal oxide composites can also be obtained by MOF precursors. Chen and coworkers have fabricated porous small size ZnCo_2_O_4_ nanoparticles (<20 nm) from a mixed zinc and cobalt-MOF, which exhibited an unexpected specific capacitance of 451 F g^−1^ at 0.5 mV s^−1^ (Chen et al., 2015). Hierarchical NiO/ZnO double-shell hollow spheres are obtained by Li and coworkers through calcining the bimetallic organic frameworks, which delivered 497 F g^−1^ at current density of 1.3 A g^−1^ (Li et al., 2016). Xu and coworkers developed a Co_3_O_4_/ZnO nano-heterostructure *via* a solid-solid conversion process, the synthesized core-shell MOFs@MOFs were used as a template with cobalt and zinc as metal sources, which demonstrated 415 F g^−1^ specific capacitance value at 0.5 A g^−1^ (Xu et al., 2016). The mixed metal oxides and the metal oxide composites as electrodes exhibit superior electrochemical performance compared with single ones. Despite these achievements, there are still large spaces to explore other metal oxide composites based on MOF precursors.

Herein, we have prepared single ZIF-67 nanocrystals first, combined it with Mn-ZIF to form ZIF-67@Mn-ZIF composite, and finally obtained Co_3_O_4_@MnO_2_ electrode material by thermal treatment. After evaluating the electrochemical performance of Co_3_O_4_@MnO_2_ electrode, we found that it exhibited excellent electrochemical properties. When the current density is 0.5 A g^−1^, the specific capacitance could achieve 413 F g^−1^, with 20 times current density increasing, it kept 41% retention of initial capacitance and good long-term cycling stability, which is a very promising electrode for use in a supercapacitor.

## Experimental

### Preparation of ZIF-67

First, 1.455g Co(NO_3_)_2_·6H_2_O and 1.642g 2-methylimidazole were separately dissolved in 40 ml methanol. Second, the two different solutions were mixed and vigorously stirred for 60 s and reacted for 24 h to complete reaction at room temperature after 24 h. Third, the purple precipitates in the bottom were collected by centrifugation with ethanol as washing solution for several times. The collected purple precipitates were dried at 80°C overnight in a vacuum drying chamber.

### Preparation of ZIF-67@Mn-ZIF

First, 0.25 g Mn(NO_3_)_2_·6H_2_O was dissolved in 50 ml ethanol. ZIF-67 obtained in the first step was well-dispersed in the above solution. Then, the mixture was transferred into a beaker flask after 20 min of continuous stirring, and the reaction temperature was 50°C and kept for 3 h in an oil bath.

### Thermal Treatment of ZIF-67@Mn-ZIF Crystals

The obtained ZIF-67@Mn-ZIF crystals could be converted to Co_3_O_4_@MnO_2_ nanomaterials through a thermal treatment in a tube furnace with air flow at 300°C for 0.5 h; the heating rate was controlled at 0.5°C·min^−1^. As a contrast experiment, the single precursors (ZIF-67) were calcined under the same thermal conditions, and the final product is Co_3_O_4_ nanomaterial.

### Material Characterizations

X-ray diffraction (XRD) patterns were measured by using monochromator Cu Kα radiation at a scanning rate of 2°•min^−1^ (PA-Nalytical X′Pert PRO). Binding energies were detected by the X-ray photoelectron spectroscopy (XPS; ESCALab250). The morphologies were obtained by scanning electron microscope (SEM) (Hitachi, SU-8000). The more detailed structures were investigated by transmission electron microscope (TEM) (JEOL, JEM-2100F), and the elements were detected by its equipped energy dispersive X-ray spectrometer (EDS). The BET surface area and pore size distribution are tested on Accelerated Surface Area & Porosimetry System (ASAP 2020, Micromeritics). XS analytical balance (Mettler Toledo; δ = 0.01 mg) is used to weigh the mass of the electrode materials.

### Electrochemical Characterizations

The electrochemical performances of the final products were accomplished by the AUTOLAB PGSTAT302N electrochemical workstation in a standard three-electrode test cell at ~25°C with 1.0 M LiOH solution as electrolyte. The Ag/AgCl (3M KCl) electrode and platinum (Pt) plate (2.5 cm × 2.5 cm × 0.2 mm) directly served as the reference electrode and counter electrode, respectively. The fabricating processes of working electrode were as follows: Co_3_O_4_@MnO_2_ materials (active electrode material, 80%) derived from ZIF-67@Mn-ZIF crystals were mixed with acetylene black (5%) and polyvinylidene difluoride (15%), which was mixed with appropriate volume N-methyl pyrrolidone solvent. The mixture was treated by ultrasonication to form a homogeneous slurry and dropped onto the graphite substrate current collector, the covered surface area is ~1 × 1 cm^2^, and then dried under vacuum condition at 120°C for 4 h to form the electrodes. For comparison, the Co_3_O_4_ materials prepared from single ZIF-67 crystals were also fabricated into electrode with the same processes.

The electrochemical performances of the fabricated electrodes were evaluated from the galvanostatic charge-discharge (GCD) and cyclic voltammetry (CV) measurements. The equation of C = [(*I* × Δ*t*)/(*m* × Δ*V*)] is applied to calculate the specific capacitance values of Co_3_O_4_@MnO_2_ and Co_3_O_4_ electrodes, where the *I* (A), Δ*t*(s), Δ*V* (V), and *m* (g) represent the discharge current, the discharge time, the potential window, the mass of active materials in the electrodes, respectively.

## Results and Discussions

**Figure 1:**
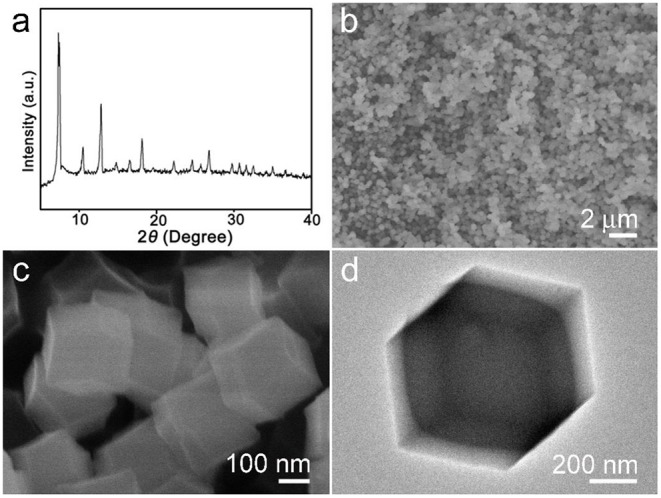
**(a)** X-ray diffraction (XRD) pattern, **(b,c)** scanning electron microscope (SEM), and **(d)** transmission electron microscope (TEM) images of synthesized ZIF-67 sample.

The synthesized products were analyzed by X-ray diffraction (XRD) first. The result is shown in Figure 1a. As can be seen from the obtained pattern, there are some strong diffraction peaks that appeared in 2θ = 7.3°, 10.4°, 12.8°, 14.8°, 16.5°, 18.1°, which can be confirmed with the sample ZIF-67 and highly consistent with reported literature (Qin et al., 2017). Figures 1b,c are the low to high magnification SEM images. The particles' morphology is uniform rhombic dodecahedral nanocrystals which were clearly monodispersed, with a diameter of about 300–500 nm. Figure 1d shows a single ZIF-67 nanocrystal with dodecahedron and solid construction. After thermal treatment, the structure collapsed (Figure S1).

**Figure 2:**
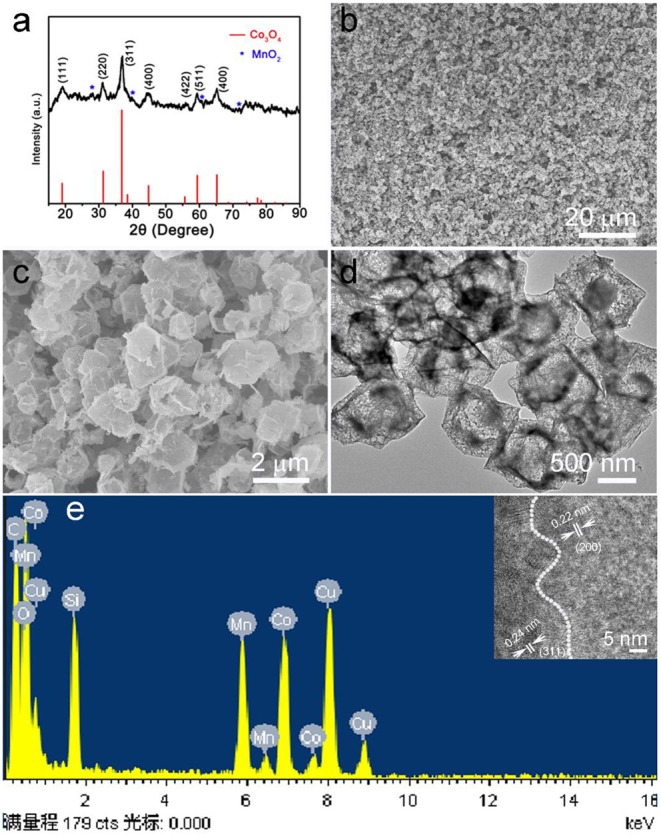
**(a)** X-ray diffraction (XRD) pattern of the products after calcinating the ZIF-67@Mn-ZIF. **(b,c)** Low magnification and enlarged scanning electron microscope (SEM) images of the Co_3_O_4_@MnO_2_. **(d)** Transmission electron microscope (TEM) image and corresponding **(e)** energy dispersive X-ray spectrometer (EDS) pattern of the Co_3_O_4_@MnO_2_ products. Inset shows the HRTEM image of Co_3_O_4_@MnO_2_.

The calcined ZIF-67@Mn-ZIF products were detected by XRD, and the result is shown in Figure 2a. The main diffraction peaks consisted of cubic phase Co_3_O_4_ (JCPDS card No. 074-2120), which is obtained by calcinating the ZIF-67 nanocrystal (Figures S2, S3). In addition, there are some other small peaks that also appeared in the pattern (marked with blue star), which could be MnO_2_ formed by Mn-ZIF (the exact components were detected by XPS, which is detailed later). Figure 2b is a low-resolution SEM image of the obtained Co_3_O_4_@MnO_2_ products, which indicated that the products can be synthesized in large scale. In the enlarged SEM image of Figure 2c, the diameter of obtained Co_3_O_4_@MnO_2_ products increased to about 800 nm; interestingly, the obtained Co_3_O_4_@MnO_2_ products are with hollow structure (Figure 2d), and the corresponding EDS result in Figure 2e is consistent with our designed concept. Co, Mn, and O elements are from Co_3_O_4_@MnO_2_ products, the existence of Cu and C signals is because the TEM grid is made of Cu substrate and carbon membrane, while the peak of Si could be an impurity that brings in the sample preparation process. Inset shows the HRTEM image of the Co_3_O_4_@MnO_2_, the *d-spacing* of 0.24 nm corresponding to the (311) lattice plane of the Co_3_O_4_ crystal, and the *d-spacing* of 0.22 nm corresponding to the (200) lattice plane of the MnO_2_ crystal (JCPDS No. 12-0716).

**Figure 3:**
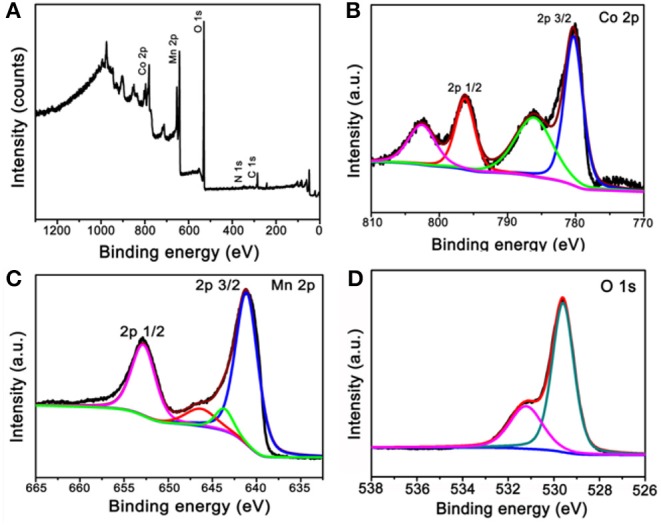
X-ray photoelectron spectroscopy (XPS) spectra of the products after calcinating the ZIF-67@Mn-ZIF. **(A)** Survey XPS spectrum and **(B–D)** high-resolution XPS spectra of Co 2p, Mn 2p, and O 1s.

The obtained calcinated products were further detected by XPS to confirm the metal oxidation states and the chemical compositions. Figure 3A is the survey spectrum of the products, which shows the core levels of Co 2p, Mn 2p, and O 1s, respectively. To get clearer information, the high-resolution XPS spectra analysis was carried out. The Co 2p's high-resolution XPS spectrum is shown in Figure 3B. The main two peaks centered at 780.3 and 796.2 eV can be appointed to the binding energies of 2p3/2 and 2p1/2 of Co(II), whereas the other two lower peaks centered at 786.1 and 802.5 eV can be appointed to the binding energies of 2p3/2 and 2p1/2 of Co(III). These results imply the Co_3_O_4_ phase in our sample and agreement with the XRD result (Yan et al., 2012; Li et al., 2013). Figure 3C is the high-resolution XPS spectrum extracted from Mn 2p. The main two peaks are centered at 641.1 and 652.7 eV; therefore, the spin-orbital splitting calculated is 11.6 eV. These results well refer to the electronic orbits of Mn 2p3/2 and 2p1/2, pointing to Mn(IV) state of the products (Sui et al., 2009). As can be seen from the high-resolution spectrum of O 1s in Figure 3D, there are two distinct components, except for the binding energy of 531.2 eV assigned to the oxygen atoms in the hydroxyl groups, the strong peak of 529.6 eV should belong to the oxygen atoms in the chemical compositions of Co_3_O_4_ and MnO_2_ (Wei et al., 2008; Xia et al., 2010). These results further proved that the chemical component of as-fabricated products is Co_3_O_4_@MnO_2_.

**Figure 4:**
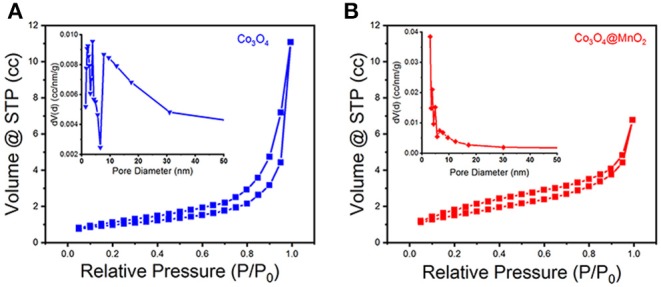
N_2_ adsorption-desorption isotherms of **(A)** Co_3_O_4_ and **(B)** Co_3_O_4_@MnO_2_ products. Inset shows their corresponding pore size distributions.

A typical IV type adsorption behavior was observed in the prepared Co_3_O_4_ and Co_3_O_4_@MnO_2_ products by the N_2_ adsorption-desorption isotherms (Figures 4A,B), which exhibit a mesoporous structure with slit type pores. The BET surface areas for the Co_3_O_4_ and Co_3_O_4_@MnO_2_ are 72.214 and 148.407 m^2^ g^−1^, a high BET surface area might be beneficial for the electrons and ions' storage and shuttle in the electrode because it provides more active sites, hence could lead to enhanced electrochemical capacity (Jiang et al., 2012). From the corresponding pore size distributions of the inset image, it can be found that the pore sizes are concentrated in 3–10 nm for Co_3_O_4_ sample and 3–6 nm for Co_3_O_4_@MnO_2_ sample. The porous structure is facilitating the electrolyte ion diffusion and transference in the course of charge and discharge processes.

**Figure 5:**
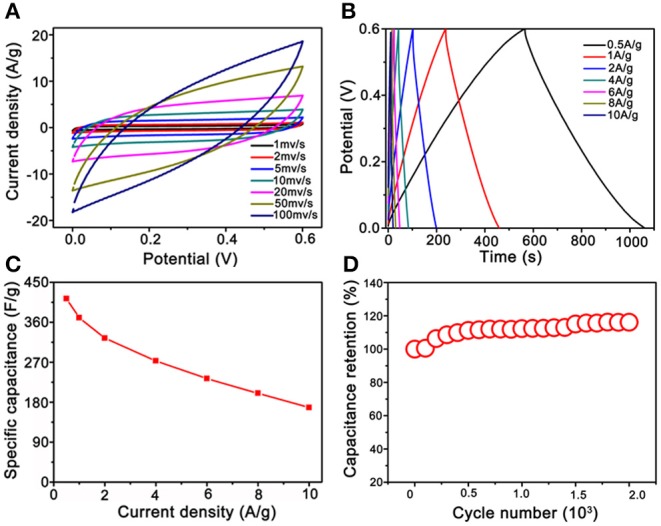
Electrochemical test results of Co_3_O_4_@MnO_2_ electrode. **(A)** Cyclic voltammetry (CV) curves at extending scan rates from 1 to 100 mV s^−1^. **(B)** CD curves under extending current densities from 0.5 to 10 A g^−1^. **(C)** Plot of the specific capacitance against different current densities. **(D)** Long-term cycling stability.

The electrochemical properties of the Co_3_O_4_@MnO_2_ electrode materials were evaluated, and the results are summarized and shown in Figure 5. Enclosed loops in Figure 5A show the electrode's CV performance at increasing scan rates from 1 to 100 mV s^−1^, unlike the pseudocapacitance behavior of single Co_3_O_4_ nanocrystals (Figure S4). The shapes of the CV curves of Co_3_O_4_@MnO_2_ indicate a typical electrical double layer capacitance (EDLC) behavior, and it retains well as the scan rate upscales to 20 mV s^−1^, demonstrating its good rate capability (Wu et al., 2015). The EDLC behavior of Co_3_O_4_@MnO_2_ ascribes to the MnO_2_ outer layer (Li et al., 2012). The slight shape deformation was observed when the scan rate achieves 50 and 100 mV s^−1^; this could be ascribed to the polarization phenomenon at high scan rate (Salanne et al., 2016). The GCD properties were evaluated at current densities from 0.5 A g^−1^ and extended to 10 A g^−1^ in the voltage range from 0 to 0.6 V vs. Ag/AgCl (3M KCl). In Figure 5B, it is clear to observe a series of good symmetric triangle shape GCD curves, revealing its good EDLC behavior; this result is consistent with CV performance. Under a series of current densities, that is, 0.5, 1, 2, 4, 6, 8, and 10 A g^−1^, the specific capacitances were calculated to be 413, 370, 324, 273, 233, 200, and 168 F g^−1^, respectively. On the contrary, single Co_3_O_4_ nanocrystal only delivers 187, 155, 108, 76, 57, and 45 F g^−1^ at 1, 2, 4, 6, 8, and 10 A g^−1^, respectively (Figure S5). Obviously, the Co_3_O_4_@MnO_2_ electrode presents better capacitance values than single Co_3_O_4_ electrode, the reason could be owing to the multicomponent and higher BET surface of Co_3_O_4_@MnO_2_ electrode that endows the more charge storage (Jiang et al., 2012). Under the extending current densities from 0.5 to 10 A g^−1^, the specific capacitance decreased from 413 to 168 F g^−1^, retaining ~41% of its initial capacitance (shown in Figure 5C). While for single Co_3_O_4_ nanocrystal, the rate capability is only 25% from 1 to 10 A g^−1^ (187 vs. 45 F g^−1^, Figure S6). After 2,000 times cycles of CV test at 20 mV s^−1^, the capacitance retention remains at 110% (shown in Figure 5D), while only 80% of single Co_3_O_4_ nanocrystal (Figure S7), indicating a good stability of Co_3_O_4_@MnO_2_ electrode. It is clear to conclude that the performance of Co_3_O_4_@MnO_2_ in connection with capacitance retention and cycling ability is much improved compared with single Co_3_O_4_.

## Conclusions

In conclusion, hollow Co_3_O_4_@MnO_2_ cubic nanomaterials were synthesized by sacrificing the ZIF-67@Mn-ZIF precursor through an uncomplicated controlled thermal treatment. The porous structure and high BET surface area endow its excellent properties as supercapacitor electrode, it presented a high specific capacitance of 413 F g^−1^ (0.5 A g^−1^) and showed a rate capability of 41% at the current density enhanced to 20 times with excellent stability, giving the impression that this hollow cubic nanomaterial possesses considerable potential as a supercapacitor electrode material.

## Data Availability Statement

The XRD datasets generated for this study can be found in the repository of ICDD, with the accession numbers of 74-2120 and 12-0716 in JCPDS card.

## Author Contributions

JW and JH conceived and designed the experiments. JX, YZ, and CX performed the experiments and analyzed the data. All authors revised and checked the draft.

### Conflict of Interest

The authors declare that the research was conducted in the absence of any commercial or financial relationships that could be construed as a potential conflict of interest.
