# Supplementary material for: Hollow Co3O4@MnO2 Cubic Derived From ZIF-67@Mn-ZIF as Electrode Materials for Supercapacitors
Source: Front Chem. 2019 Dec 13;7:831. doi: 10.3389/fchem.2019.00831 (PMC6923731; doi:10.3389/fchem.2019.00831)
Supplement: Supplementary file 1 [file Table_1.docx]

**Supporting information**

Hollow Co_3_O_4_@MnO_2_ cubic derived from ZIF-67@Mn-ZIF as electrode materials for supercapacitors

Jiani Xu,^†^ Chaoting Xu,^†^ Yanhong Zhao,^†^ Jianghong Wu*^‡§^ and Junqing Hu^‡^

**^‡^** College of Health Science and Environmental Engineering, Shenzhen Technology University, Shenzhen, Guangdong 518118, PR China

**^†^** State Key Laboratory for Modification of Chemical Fibers and Polymer Materials, College of Materials Science and Engineering, Donghua University, Shanghai 201620, China

^§^ College of Materials Science and Engineering, Changsha University of Science & Technology, Changsha, Hunan 410114, PR China

***Correspondence:**wujianghong@sztu.edu.cn


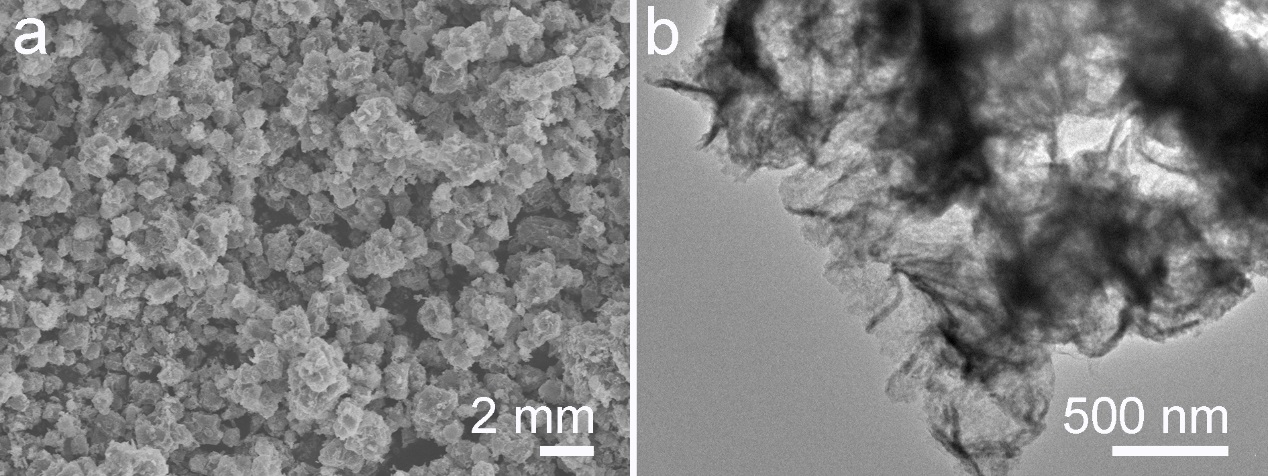


Figure S1 (a) SEM image and (b) TEM image of Co_3_O_4_

**
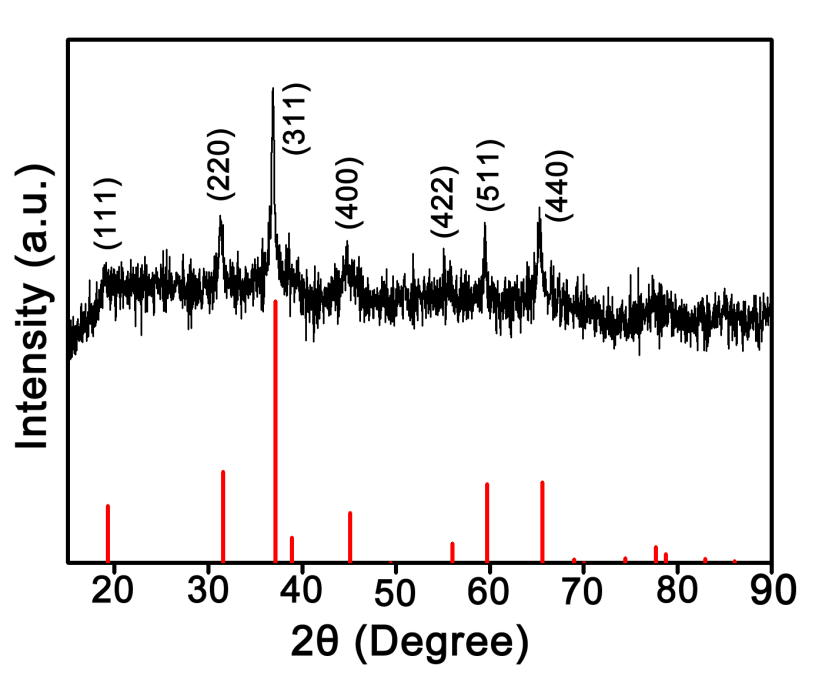
**

Figure S2 XRD pattern of Co_3_O_4_ obtained from calcining the ZIF-67, the JCPDS Card No. is 74-2120.

**
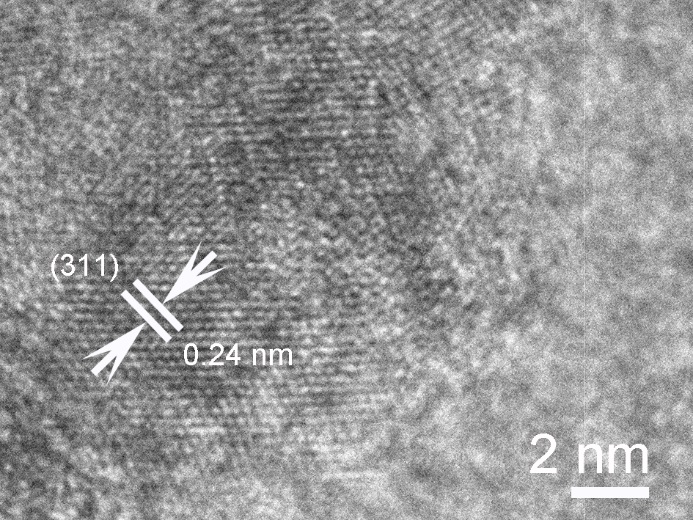
**

Figure S3 HRTEM image of Co_3_O_4_, the *d-spacing* of 0.24 nm corresponding to that of the (311) lattice planes of the crystal.

**
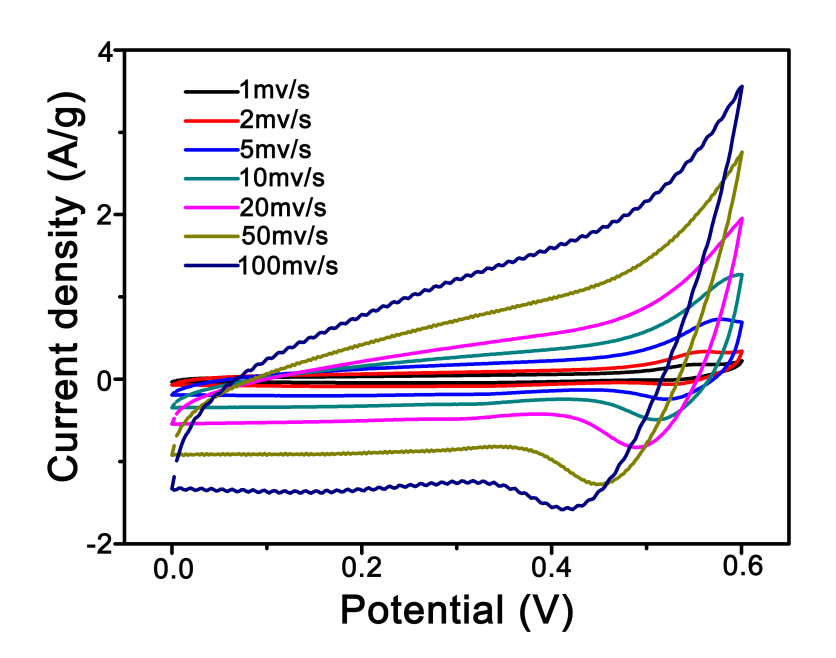
**

Figure S4 CV curves for as-synthesized single Co_3_O_4_ nanocrystal.

**
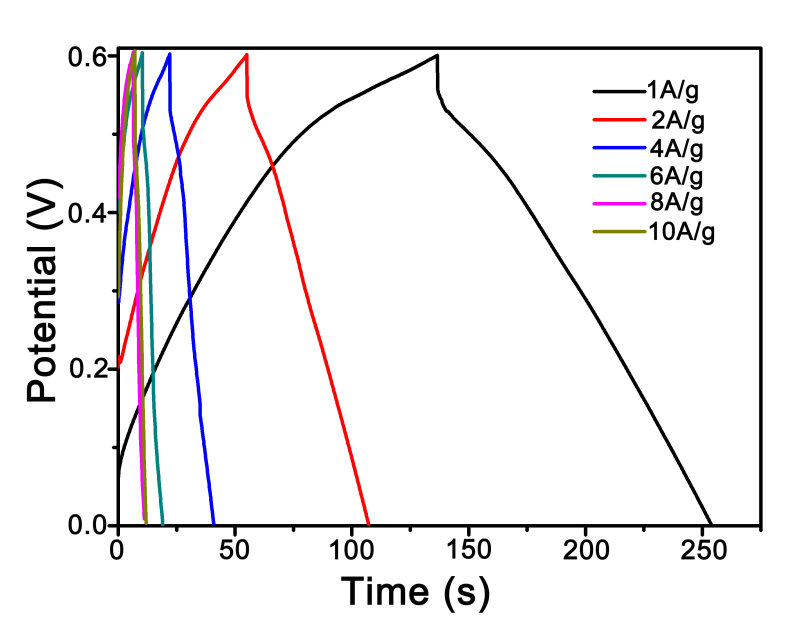
**

Figure S5 Galvanostatic charge-discharge curves for as-synthesized single Co_3_O_4_ nanocrystal. The specific capacitances at different current densities were calculated to be 187, 155, 108, 76, 57, and 45 F g^-1^ at the current density of 1, 2, 4, 6, 8 and 10 A g^-1^, respectively.

**
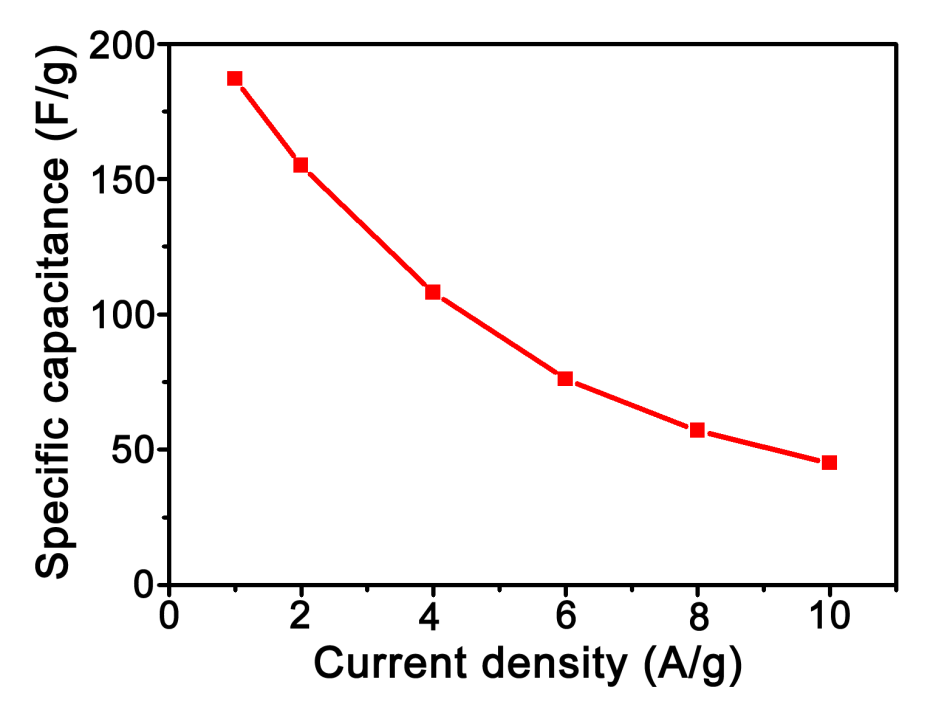
**

Figure S6 Plot of the specific capacitance of Co_3_O_4_ nanocrystal against the different current densities. The rate capability is 25% from 1 to 10 A g^-1^ (187 vs. 45 F g^-1^).

**
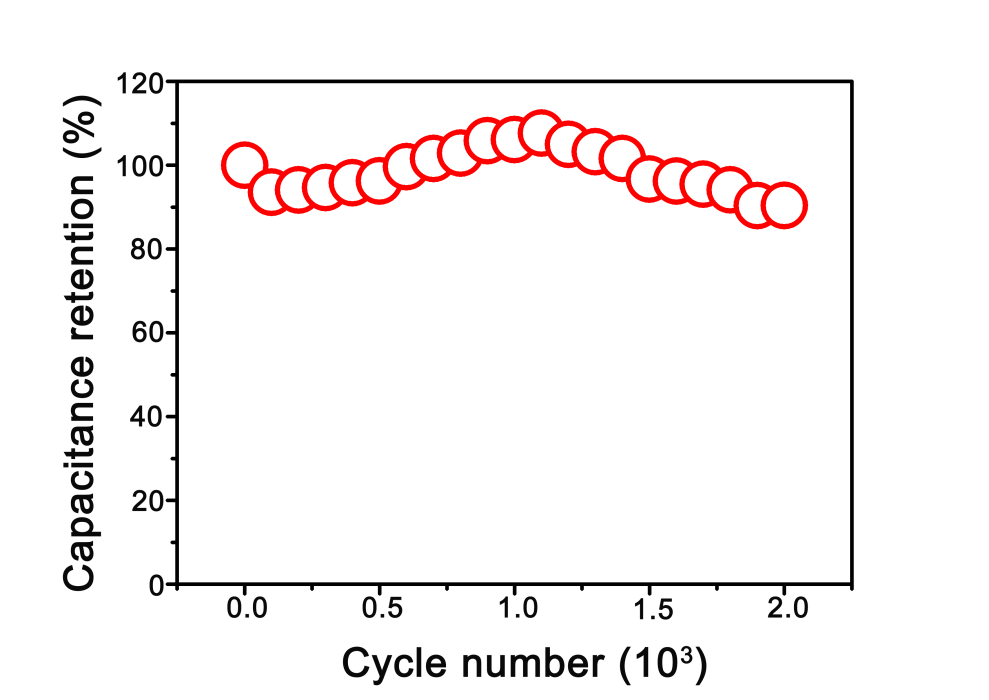
**

Figure S7 Cycling stability test of Co_3_O_4_ nanocrystal at scan rate of 20 mV s^-1^. In the first 1000 times cycling, the specific capacitance is increasing, while along with the times increasing continue, the specific capacitance is decreasing gradually, finally keep 80% of initial capacitance after 2000 times cycling.
